# Understanding the effects of COVID-19 on patients with diabetic nephropathy: a systematic review

**DOI:** 10.1097/MS9.0000000000002053

**Published:** 2024-04-17

**Authors:** Samar M. Altoukhi, Mariam M. Zamkah, Reman A. Alharbi, Shatha K. Alghamdi, Lama S. Aldawsari, Muyassar Tarabulsi, Hisham Rizk, Yousif Sandokji

**Affiliations:** a College of Medicine; Departments ofb Medical Microbiology and Parasitology; c General Surgery, Faculty of Medicine, University of Jeddah, Jeddah, Saudi Arabia

**Keywords:** ACE2, diabetic kidney disease, diabetic nephropathy, SARS-COV-19

## Abstract

**Background:**

Diabetic nephropathy is one of the consequences of diabetes mellitus that causes a continuous decline in the eGFR. After the COVID-19 pandemic, studies have shown that patients with diabetic nephropathy who had contracted COVID-19 have higher rates of morbidity and disease progression. The aim of this study was to systematically review the literature to determine and understand the effects and complications of SARS-CoV-2 on patients with diabetic nephropathy.

**Materials and methods:**

The authors’ research protocol encompassed the study selection process, search strategy, inclusion/exclusion criteria, and a data extraction plan. A systematic review was conducted by a team of five reviewers, with an additional reviewer assigned to address any discrepancies. To ensure comprehensive coverage, the authors employed multiple search engines including PubMed, ResearchGate, ScienceDirect, SDL, Ovid, and Google Scholar.

**Results:**

A total of 14 articles meeting the inclusion criteria revealed that COVID-19 directly affects the kidneys by utilizing ACE2 receptors for cell entry, which is significant because ACE2 receptors are widely expressed in the kidney.

**Conclusion:**

COVID-19 affects kidney health, especially in individuals with diabetic nephropathy. The mechanisms include direct viral infection and immune-mediated injury. Early recognition and management are vital for improving the outcomes.

## Introduction

Highlights

- Individuals with diabetes and diabetic nephropathy are at a higher risk of poor renal outcomes in COVID-19.
- COVID-19-associated nephropathy is associated with increased mortality and end-stage kidney disease.
- Mechanisms such as direct viral infection, immune-mediated injury, and thrombotic microangiopathy contribute to kidney injury in COVID-19 patients.
- Monitoring kidney function, early recognition, and management of COVID-19-associated nephropathy are crucial for improving prognosis in COVID-19 patients with underlying kidney conditions.
- Understanding the role of ACE2 receptors and microRNAs in the interaction between SARS-CoV-2 and kidney cells is crucial for future research and therapeutic development to improve outcomes in COVID-19 patients with kidney complications.

The COVID-19 pandemic has had a significant impact on global health, with millions of confirmed cases and deaths occurring worldwide. COVID-19 is a highly infectious respiratory illness caused by SARS-CoV-2. The virus was first identified in Wuhan, China, in December 2019 and has since spread rapidly worldwide^1^. COVID-19 is associated with a range of complications, including acute respiratory distress syndrome, sepsis, and multi-organ failure^2^. The impact of COVID-19 on patients with pre-existing medical conditions, such as diabetes, is a growing concern. Despite the complications caused by COVID-19, it is noteworthy that recent studies have found a significant decrease in hospitalization rates among COVID-19 patients, with viral vector vaccines demonstrating a notable reduction of 67%^3^.

Diabetic kidney disease (DKD) is a common complication of diabetes that affects the kidney. It is characterized by the progressive loss of kidney function and the development of albuminuria, which is the presence of excess protein in the urine. DKD is a leading cause of end-stage renal disease (ESRD) and is associated with increased morbidity and mortality. The prevalence of DKD is increasing worldwide, with an estimated 40% of patients with diabetes developing DKD^4^.

The combination of COVID-19 and DKD poses specific challenges, as SARS-CoV-2 has been shown to target and bind to ACE2 receptors that are highly expressed in the kidneys^5^. This raises the possibility of kidney damage in individuals with DKD who have contracted the virus. Previous studies have indicated an increased risk of fatality, greater disease severity, and worse outcomes in individuals with DKD and diabetes who are infected with COVID-19^5,6^. Understanding the mechanisms underlying the interaction between SARS-CoV-2, diabetes, and kidney disease is crucial for identifying potential therapeutic targets and improving patient outcomes^7^.

This systematic review aimed to explore the existing literature on the relationship between COVID-19 and DKD, focusing on the mechanisms by which COVID-19 exacerbates kidney damage in individuals with diabetes. We will examine studies that investigate the expression of ACE2, the impact of COVID-19 on renal function, and the potential pathways involved in kidney injury. Additionally, we will explore the role of comorbidities such as hypertension and obesity on the severity of COVID-19 in patients with DKD. Through a comprehensive analysis of the available evidence, we sought to gain insight into the complex interplay between SARS-CoV-2 infection, diabetes, and kidney disease. By elucidating the underlying mechanisms, this review aims to contribute to the development of effective treatments and interventions for individuals with DKD who have contracted COVID-19.

## Methodology

### Literature search

We developed a research protocol in accordance with the PRISMA statement, which included the study selection process, search strategy, inclusion/exclusion criteria, and a plan for extracting data. This study was registered in the International Prospective Register of Systematic Reviews (PROSPERO)(ID:CRD42023455409). It has also been reported in line with AMSTAR (Assessing the methodological quality of systematic reviews) Guidelines. The search was initiated in January 2023 and ended in October 2023. We conducted a systematic search using six search engines: PubMed, ResearchGate, Science Direct, SDL, Ovid, and Google Scholar. The keywords used were (COVID-19 OR Coronavirus OR SARS-CoV-2) AND (Diabetic nephropathy OR Diabetic Kidney Disease), and the search results were limited to studies published between 2019 and 2023.

### Study selection

**Figure 1:**
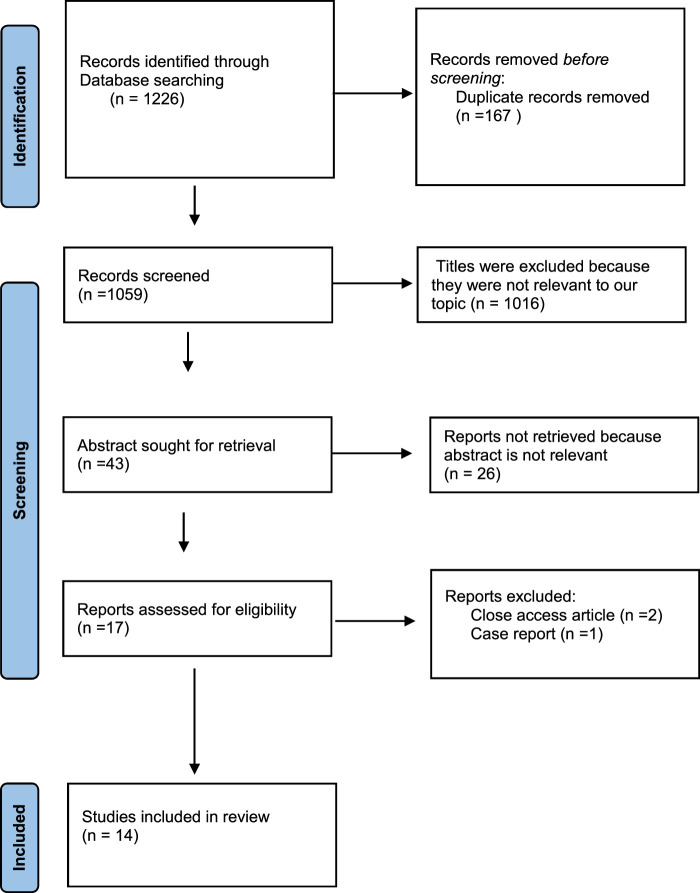
PRISMA flow chart.

English language studies that assessed the impact of COVID-19 on diabetic nephropathy were included in this systematic review. Studies that met the inclusion criteria included randomized controlled trials, case series, cohort, retrospective, and prospective designs. Studies that lacked access to the full text, meta-analyses, or case reports were excluded. Studies on unrelated patient groups, interventions, or outcomes were also excluded. As part of our comprehensive approach to identifying relevant primary articles, we utilized existing systematic reviews related to our research topic as a source for collecting and inventorying research. These systematic reviews served as valuable resources for identifying the original studies that met our inclusion criteria. We carefully reviewed the primary articles cited within these systematic reviews to analyze and incorporate them into our research. A total of 14 articles were included, as shown in (Fig. 1).

### Data extraction

Full-text articles were retrieved and screened by five independent investigators, and one investigator was responsible for any discrepancies or disagreements. Relevant information, such as authorship, country of origin, study design, sample size, and publication year, was collected from the articles. The results or findings related to the impact of COVID-19 on diabetic nephropathy and any additional relevant information were extracted. A summary of the included studies is shown in (Table 1).

**Table 1:** A summary of included studies

Author and year	Population	Outcomes
Richard E. Gilbert MD, PhD a, 18 July 2020^8^	Patients with COVID-19 and diabetic kidney disease	The results of the kidney biopsies from DKD patients and healthy individuals showed Increased ACE2 messenger RNA in the diabetic kidney may increase the risk and/or severity of kidney infection with SARS-CoV-2 in the setting of COVID-19 disease
Maria F. Gomez and Kumar Sharma, October 7 2020^9^	Healthy living donors (LDs), patients with diabetic kidney disease (DKD), and COVID-19 patients that required hospitalization (COV)	The potential role of the ACE2 receptor and other receptor networks in the development of kidney disease, includes an imbalance in the renin-angiotensin-aldosterone system, inflammation, and potential direct viral injury to the kidney. A better understanding of these receptor networks may lead to new treatments for kidney disease in people with diabetes and COVID-19
Yang, Yang, 20 October 2022^10^	Patients with diabetic kidney disease complicated with COVID-19	The article summarized the relationship between COVID-19 and DKD, and the possible mechanism of elevated blood glucose after COVID-19 vaccination
Gabriel Giannini, 30 September 2022^11^	Patients with COVID-19-associated nephropathy (COVAN) who were admitted to a single tertiary care centre in the United States	Patients with COVID-19-associated nephropathy (COVAN) had a higher risk of all-cause mortality and end-stage kidney disease (ESKD) compared to patients with COVID-19 without nephropathy. 47.1% of patients with COVAN developed ESKD within 6 months of COVID-19 diagnosis, compared to 3.2% of patients with COVID-19 without nephropathy. The study also found that Black patients with COVAN had a higher risk of ESKD compared to non-Black patients. The authors concluded that COVAN is associated with poor renal outcomes and that early recognition and management of COVAN may improve the prognosis of patients with COVID-19
Alok K. Paul, 13 January 2022^12^	Patients with diabetes and COVID-19	The ACE2 depletion and angiotensin II interaction, which result in oxidative stress and blood vessel constriction, are the common inflammatory mechanisms in COVID-19 and diabetes. According to the theory, COVID-19 and diabetes together may synergistically enhance oxidative stress, which may then result in serious side effects including end-stage renal failure and mortality. Antioxidants like vitamin D and flavonoids may shield diabetics against the problems of COVID-19
Diane Mourad, 21 July 2021^13^	Patients with diabetic nephropathy disease complicated with COVID-19	Diabetic patients, especially those with Diabetic Nephropathy, are at high risk for severe COVID-19. The underlying mechanisms are not fully understood, but two factors have been hypothesized: ACE2 up-regulation caused by certain medications used by diabetic patients, and the involvement of NRP-1 in viral entry. Other factors such as AGEs, mitochondrial glutathione, Vitamin D, and dipeptidyl peptidase 4 may also play a role but require further investigation
Gyanendra Kumar Sonkar, 21-December-2022^14^	Patients with diabetes and COVID-19	Diabetic patients with COVID-19 face challenges like pneumonia, hospitalization, intubation, and early mortality. Those with both complications and COVID-19 require intensive treatment and have worse outcomes than those with just diabetes. Diabetic nephropathy and COVID-19 have a complex relationship. COVID-19 can cause acute kidney injury, with a high risk of renal replacement therapy and mortality. The SARC‑CoV‑2 virus targets renal cells through the ACE2 receptor, leading to acute tubular injury, hypouricemia, uricosuria, respiratory decompensation, and severe disease
Swayam Prakash Srivastava, 30 July 2021^15^	Diabetic and diabetic kidney disease patients with COVID-19	About 20–30% of diabetic patients develop diabetic nephropathy, which worsens COVID-19. Clinical signs of COVID-19-associated nephropathy include glomerular nephrosis, AKI, and CKD. MicroRNAs show promise as therapeutic targets for kidney disease and can inhibit SARS-CoV-2 infection. ACE2 and TMPRSS2 are receptors involved in viral uptake, with ACE2 playing a role in kidney injury-associated inflammation and regulated by specific microRNAs. LncRNAs and microRNAs interact to influence disease phenotypes in diabetic kidney disease
Juan Alonso Leon-Abarca, 10 Nov 2020^16^	COVID-19 patient associated with, Chronic Kidney Disease, diabetic nephropathy	Patients with diabetic kidney disease were 10.7% more likely to be infected with SARS-CoV-2 compared to CKD patients, doubled their rate of COVID-19 pneumonia (107.9% more) The adjusted prevalence analysis relieved that there was an 87.9% higher probability of developing COVID-19 pneumonia in patients with diabetic nephropathy
S. Charles Bronson, 21 May 2021^17^	Patients with diabetic kidney disease complicated with COVID-19	Diabetic patient with Pre-exiting diabetic nephropathy and chronic kidney disease CKD have the risk to develop an acute exacerbation of CKD during COVID-19 infection
Martin Schiller, 15 July 2021^18^	75 COVID-19 patients	Diabetic nephropathy or obesity plus diabetes are important risk factors for mortality
Fahad Abdulaziz Al-Muhanna, 6 May 2022^5^	Diabetic Patients with Covid-19	Diabetic patients had a higher likelihood of needing ICU admission for severe COVID-19 compared to non-diabetic patients
Ruman Basra, 31 December 2021^6^	Individuals with COVID-19 and diabetes	Microvascular complications in patients with diabetes are linked to worse prognosis in COVID-19
Luis D’Marco, 06 May 2020^7^	DKD patients with COVID-19	COVID-19 patients with diabetic kidney disease (DKD) should be closely monitored for worsening disease stage, as it may indicate a higher risk of severe outcomes like renal replacement therapy and fatality

## Results

SARS-CoV-19 causes multiple organ damage; however, the kidney is more susceptible to damage due to the expression of ACE2 receptors, which the virus uses to gain entry into the cells^5^. A study published on 18 July 2020, investigated the expression of ACE2 in 49 biopsies of patients with diabetic kidney disease and found that the expression of ACE messenger RNA was significantly increased by a factor of two when compared to healthy control subjects. This finding is particularly concerning because SARS-CoV-2 specifically targets and binds to the ACE2 receptor. Therefore, individuals with diabetic kidney disease who exhibit increased ACE expression may be at a higher risk of developing acute kidney injury (AKI) if they contract COVID-19. Additionally, the interaction between the virus and ACE2 may lead to an imbalance in the renin-angiotensin system, a complex system that regulates blood pressure and fluid balance in the body. This imbalance may explain the cause of kidney injury in patients with DKD infected with SARS-CoV-2^8^.

In two studies published in 2022^5,6^, the authors reported that individuals with diabetes mellitus and DKD in COVID-19 cases were associated with an increased risk of fatality, greater disease severity, and worse outcomes. One study gathered data from two hospitals, including 1561 individuals who had contracted COVID-19. The results of the study indicated that patients with diabetes (9.8%) were more likely to require admission to the ICU than patients without diabetes. These findings suggest that diabetes and poor glycemic control are significant risk factors for severe COVID-19 illness and are correlated with worse outcomes^5^. Furthermore, microvascular complications in patients with diabetes are linked to a worse prognosis in COVID-19^6^.

A systematic review^7^ that analyzed multiple studies, two of which were relevant to the present topic^19,20^, reported several potential mechanisms by which COVID-19 can exacerbate kidney damage. These include cytokine storm syndrome, direct viral injury to renal tubular cells, or sepsis-related pathways^19^. While COVID-19-related kidney damage is often acute, some patients may experience haematuria, macroalbuminuria, or proteinuria, which may be associated with the endothelial dysfunction observed in these individuals^20^.

Individuals with DKD who contract COVID-19 should be closely monitored for worsening disease staging, as this may indicate a heightened risk of adverse outcomes, such as renal replacement therapy and fatality^7^. It has also been confirmed that individuals with diabetic nephropathy who contract COVID-19 are susceptible to worsening kidney disease. Furthermore, diabetic patients without diabetic nephropathy are at risk of developing AKI during hospitalization^17^.

A study published in October 2020 investigated the relationship between SARS-CoV-2 infection, diabetes, and kidney disease. Using single-cell RNA sequencing and in-situ hybridization, the researchers examined the expression of ACE2 and other factors involved in SARS-CoV-2 entry into kidney cells from 18 healthy donors, 44 patients with diabetic kidney disease, and 13 COVID-19 patients requiring hospitalization was determined. They found that ACE2 expression levels were higher in patients with diabetic kidney disease, making them more susceptible to severe COVID-19 outcomes because of two potential interactions with SARS-CoV-2. First, the enhancement of viral infection pathways in patients with diabetic kidney disease may explain why this group was more vulnerable to the virus. Second, if the virus infects ACE2 proximal tubular epithelial cells and further activates pathways already upregulated in diabetes, it can lead to kidney damage. This article also highlights the importance of considering other receptor networks and pathways involved in kidney disease development, such as the ACE homologue ACE, which can contribute to the development of kidney disease by promoting vasoconstriction and inflammation. The NLRP3 inflammasome is another pathway implicated in kidney injury as it can trigger the release of pro-inflammatory cytokines. Finally, the Toll-like receptor pathway is involved in the innate immune response and may contribute to the development of inflammation and fibrosis in the kidney. The authors suggested that understanding these receptor networks may have significant implications for the development of effective treatments for kidney disease in individuals with diabetes and COVID-19^9^.

A review study published on 20 October 2022^10^ examined the correlation between COVID-19 and DKD and the potential mechanisms for elevated blood glucose levels following vaccination. A study conducted in a German community hospital analyzed a cohort of 75 COVID-19 patients. The effects of various comorbidities on the COVID-19 disease course were assessed in this study, and diabetes was found to be a significant risk factor for extended hospital stay and higher mortality. The worst outcomes were observed in patients with established diabetic nephropathy^18^. Specifically, DKD patients are 10.7% more likely to contract SARS-COV-2 and twice as likely to develop COVID-19 pneumonia, and have higher rates of intubation and case-fatality than patients with chronic kidney disease, which may be linked to a pro-inflammatory state and immune dysfunction in DKD patients^16^. This was also mentioned in a review article^5^ published by Fahad Abdulaziz Al-Muhanna in 2022.

Additionally, more than half of maintenance haemodialysis patients with COVID-19 also have underlying DKD, and these individuals have been found to be at higher risk of ICU admission and mortality^21^. Taken together, patients with DKD are increasingly recognized as a particularly vulnerable group for SARS-CoV-2 infection, with a higher likelihood of progressing to severe COVID-19.

Moreover, this study^18^ investigated the potential association between DKD and COVID-19 through the comprehensive elucidation of five plausible factors, starting with the Renin-Angiotensin-Aldosterone System (RAAS), which plays a key role in the development of DKD. ACE2, a therapeutic target, converts Angiotensin II (Ang II) to Angiotensin 1-7 (Ang 1-7), which has beneficial effects in countering the negative effects of Ang II. Studies have shown that patients with DKD have a high ACE/ACE2 ratio, which leads to decreased kidney function and injury. SARS-CoV-2, the virus causing COVID-19, enters cells through ACE2 and triggers an immune response that exacerbates renal injury in patients with DKD. The expression of ACE2 is reduced upon SARS-CoV-2 infection, leading to the accumulation of Ang II and increased aldosterone secretion, which can have various harmful effects. Downregulation of the ACE2/Ang 1-7/Mas receptor axis and amplification of the ACE/Ang II/AT1R axis are detrimental to patients with DKD and COVID-19^22–24^.

Additionally, according to a cross-sectional study in China, increased dipeptidyl peptidase 4 (DPP4) activity is closely linked to type 2 diabetes mellitus-related DKD. The study found that as the DPP4 quartile increased, the levels of oxidative stress, interleukin-6 (IL-6), and C-reactive protein (CRP) also increased^25^. It was also found that inhibiting AMP-activated protein kinase (AMPK) activation in renal cells is one of the mechanisms underlying COVID-19 infection^15^. DPP4 inhibitors, such as linagliptin and alogliptin, can significantly reduce the activity of advanced glycation end products (AGE) and the AGE receptor axis, thus reducing renal damage caused by inflammation, proteinuria, and oxidative stress in individuals with type 1 and type 2 diabetes mellitus. In diabetics, the increased level of the soluble form of the AGE receptor in circulation is linked to inflammatory markers and proteinuria, which can serve as a biomarker for vascular injury in type 2 diabetes mellitus^21,26–28^. Accordingly, inhibition of DPP4 and up-regulation of AMPK may have a protective effect, highlighting the negative impact of DPP4 and the potential positive impact of AMPK in individuals with diabetes who have contracted COVID-19^15^.

Finally, increased neuropilin-1 (NRP-1) in diabetic nephropathy facilitates viral entry into tissues^15^. Therefore, the up-regulation of neuropilin-1 (NRP-1) is associated with worsening of renal function, as evidenced by the strong correlation between NRP-1 expression and levels of serum creatinine and urea in severe COVID-19 patients. After infection, consumption of NRP-1 may lead to impaired podocyte function and exacerbate DKD^13,29,30^. Furthermore, studies have revealed that short-term erythropoietin treatment can reverse NRP-1 expression, decrease proteinuria, and protect podocytes from damage caused by AGE, indicating that decreased NRP-1 expression is characteristic of DKD^31,32^.

To Summarize, article^10^ emphasizes that the connection between COVID-19 and DKD is intricate and bidirectional, with RAAS activation, chronic inflammation, and endothelial dysfunction being the key factors connecting the two conditions. Endothelial dysfunction leads to a procoagulant and anti-fibrinolytic state, whereas SARS-CoV-2 disrupts the glucose-insulin axis, causing oxidative stress and worsening DKD. DPP4 inhibitors have a protective effect on organs by reducing inflammation; however, the role of NRP-1 requires further investigation. Vaccines have helped control the spread of COVID-19, despite some adverse reactions that can be mitigated through early detection. More research is needed to understand the relationship and prognosis between DKD and COVID-19 owing to the lack of relevant experimental models.

Additionally, a study published in 2020 by Giannini investigated the renal prognosis of COVID-19-associated nephropathy (COVAN) in patients from a tertiary care centre in the United States. This study compared the clinical outcomes of patients with COVAN to those of patients without nephropathy. Although the research did not show a direct correlation between diabetes and COVID-19-associated nephrology, 27.9% of the patients had diabetes mellitus as a comorbidity. This article discusses several possible mechanisms by which SARS-CoV-2 may cause kidney injury, including direct viral infection of the kidney cells, immune-mediated injury, and thrombotic microangiopathy. The authors also noted that certain populations, such as those with APOL1 variants, may be at an increased risk of developing COVAN. Moreover, researchers found that patients with COVAN had a higher risk of all-cause mortality and end-stage kidney disease (ESKD) than patients without nephropathy. Furthermore, the study discovered that 47.1% of patients with COVAN developed ESKD within 6 months of COVID-19 diagnosis compared to only 3.2% of patients without nephropathy. The study also revealed that black patients with COVAN had a higher risk of ESKD than non-black patients. The authors concluded that COVAN is associated with poor renal outcomes and that early recognition and management of COVAN may improve the prognosis of patients with COVID-19. Overall, this study highlights the importance of monitoring kidney function in patients with COVID-19, particularly those with COVAN, to identify and manage potential complications^11^.

Furthermore, Alok K. Paul and colleagues’ subsequent study shed light on the effects of COVID-19 on diabetic nephropathy. According to a previous study, COVID-19 can worsen kidney damage in people with diabetes because they both have inflammatory pathways that may increase oxidative stress. Based on the findings of this study, the combination of COVID-19 and comorbid conditions such as diabetes could synergistically increase oxidative stress, which could result in mortality and end-stage renal failure. Antioxidants have been suggested as potential defenses against comorbidities associated with COVID-19, such as diabetic nephropathy. In a small number of patients, clinical trials have shown that antioxidant therapy is effective in reducing COVID-19 symptoms. The study recommended further research into the therapeutic or dietary efficacy of antioxidants, such as vitamin D and flavonoids, in the context of COVID-19 with diabetes as a comorbidity^12^.

A narrative review^13^ hypothesized that several factors are involved in the mechanism of severe COVID-19 disease progression in DN patients with diabetic nephropathy. The first factor is ACE, which is the primary host receptor of SARS-CoV-2. This virus entry is facilitated by the binding of the SARS-CoV-2 spike receptor-binding domain (RBD) domain to ACE2^33^. Hospitalized COVID-19 patients who are diabetic are using ACEIs and ARBs as a first-line agent for their nephroprotective effects; however, their administration leads to up-regulation of ACE2, which could potentially enhance the virus’s ability to enter the body^34^. In addition, a reduction in ACE2 caused by SARS-CoV-2 binding can increase the ACE/angiotensin II signalling pathway and related pathologies^35^. Therefore, COVID-19 is characterized by ACE2 depletion, probably playing a key role in the devastating cytokine storm characterizing this disorder^36^. Moreover, patients with diabetes and kidney disease were found to have increased ACE2 expression in proximal tubular epithelial cells, and the expression of ACE2 mRNA levels was notably unregulated compared to that in normal kidneys^9^. This elevated expression in the kidneys of diabetic patients may increase the susceptibility and/or severity of kidney infection with SARS-CoV-2 in the setting of COVID-19^8^.

In addition, A systemic review^14^ published in 2020 by Gyanendra Kumar Sonkar summarized the latest advances, updates, and discoveries related to the impact of COVID-19 on individuals with diabetes and their microvascular complications Focusing on diabetic nephropathy and COVID-19, there is evidence indicating a complex pathophysiology between them. Acute kidney injury is a serious complication of COVID-19 due to the high risk of renal replacement therapy and mortality. The mechanism of SARC-CoV-2 associated with the AKI is attacking renal cells through the cell surface receptor (ACE2), besides; acute tubular injury is the most common aetiology, which causes hypouricemia and uricosuria that is correlated with respiratory decompensation and disease severity. Another study suggested that 27% of AKI patients have diabetic nephropathy^37–40^.

Moreover, a paper published in 2021 by Swayam Prakash Srivastava^15^ describes COVID-19 severity in diabetes and diabetic kidney disease. miRNAs have shown great potential as therapeutic targets for kidney disease and have also been found to inhibit SARS-CoV-2 infection by their protein-expressing genes related to structural and nonstructural proteins^41^. However, there is limited knowledge regarding the specific mechanisms by which miRNAs regulate SARS-CoV-2 gene expression in kidney cells to regulate viral DNA amplification. ACE2 and TMPRSS2 are receptors involved in the cellular uptake of SARS-CoV-2^42,43^. ACE2 is highly expressed in various tissues, including the kidneys. Disease severity is linked to the binding affinity of the virus to host cells. ACE2 plays a crucial role in the inflammation associated with kidney injury and is regulated by certain miRNAs^43^. Long non-coding RNAs (lncRNAs) and microRNAs interact to influence the phenotypes of DKD^44^. Studies have shown differential expression of lncRNAs in lung tissue from COVID-19 patients, including MALAT1 and NEAT1^45^. These ncRNAs are associated with inflammation and viral replication^46,47^. The circular RNA circACTR2 has also been implicated in inflammation and DKD pathogenesis^48^. However, further research is required to understand their specific roles in patients with DKD and COVID-19.

### HbA1c, medications, and other affecting factors

Chronic kidney disease (CKD) impacts the adult population worldwide, and its prevalence tends to increase among older adults^16^.

Due to the frequent coexistence of extreme obesity and hypertension in diabetic individuals, it remains uncertain whether diabetes mellitus alone contributes to an increased susceptibility to the adverse outcomes and mortality associated with COVID-19^49^. It was also reported that individuals with diabetes who were using ACE inhibitors and ARBs experienced elevated levels of ACE2 expression. This heightened expression of ACE2 can potentially facilitate the infection of COVID-19. Furthermore, the continued use of ACE2 inhibitors in severe cases of COVID-19 has been associated with an increased risk of mortality^16,50,51^. CKD, male gender, old age, and high blood pressure are significant factors that are forecasters of covid-19 death^52^.

Diabetic patients with COVID-19 face challenges like pneumonia, hospitalization, intubation, and early mortality. While the exact reasons behind increased mortality in COVID-19 patients with diabetes are still being investigated, several factors are likely at play. These include the duration of the diabetic condition, age, gender, race, and the individual’s ability to manage blood sugar levels^14^.

About 20–30% of diabetic patients develop diabetic nephropathy, which worsens COVID-19, because, over time, hyperglycaemia damages the glomeruli leading to potential kidney failure. Clinical signs of COVID-19-associated nephropathy include glomerular nephrosis, AKI, and CKD with symptoms, such as fluid and electrolyte imbalance, hypertension, acid-base derangements, and oedema^15^.

Additionally, there have been reports linking higher levels of glycosylated haemoglobin (HbA1c) with a 60% increased risk of hospitalization and severity of pneumonia in bacterial infections. Hyperglycaemia is considered an independent factor that heightens the risk of death and worsens the prognosis^16^.

Another Study revealed significant variation in the expression of the ACE2 gene in kidney biopsies taken from patients with diabetic kidney disease in comparison to biopsies from healthy individuals; however, no differences in transcript abundance were observed between individuals who received medications that block the renin-angiotensin-aldosterone system (RAAS) and those who did not, including patients treated with ACE inhibitors, ARBs, DRIs, diuretics, or MRAs. Furthermore, there was no correlation found between ACE2 copy number and either glycated haemoglobin levels or estimated glomerular filtration rate^8^.

Several additional risk factors can contribute to the development or worsening of diabetic nephropathy in the context of COVID-19. Research has indicated that African patients with a genomic risk in the apolipoprotein L1 (APOL1) gene are more likely to experience collapsing glomerulonephritis when infected with the SARS-CoV-2 virus. Furthermore, among the studied patients, 19 out of 56 (44%) had pre-existing CKD. The presence of certain comorbidities also increases the risk of collapsing glomerulonephritis associated with SARS-CoV-2, including hypertension in 33 patients (76.7%), obesity in 26 patients (60%), smoking in 2 patients (7%), and diabetes mellitus in 12 patients (27.9%). Notably, 81.4% of the patients had at least one of these conditions^11^.

As Schiller and colleagues reported, diabetic nephropathy and the combination of obesity with diabetes significantly increase COVID-19 mortality rates, exceeding 70%. Additionally, their study points out that other factors, including older age, congestive heart failure, and chronic kidney disease, notably influence COVID-19 disease severity and survival rates. These findings highlight the importance of considering differences in comorbidities when assessing COVID-19 risk and prognosis. This article also found that diabetic nephropathy and combined obesity with diabetes were associated with increased COVID-19 mortality, the study did not delve into the potential relationship between HbA1c levels and COVID-19 outcomes. The reported HbA1c levels were similar between survivors and non-survivors (HbA1c 6.34% vs. 6.36%, *P* value=0.7760). It’s important to consider that a single HbA1c measurement might not reflect recent glycemic control fluctuations, which could be relevant to COVID-19 outcomes^18^.

## Conclusion

Studies have shown that COVID-19 can lead to poor renal outcomes, particularly in individuals with comorbidities such as diabetes and diabetic nephropathy. COVAN is associated with a higher risk of mortality and ESKD. Mechanisms such as direct viral infection, immune-mediated injury, and thrombotic microangiopathy contribute to kidney injury in COVID-19 patients. Monitoring kidney function and early recognition and management of COVAN are crucial for improving prognosis. Additionally, COVID-19 can exacerbate kidney damage in individuals with diabetes through shared inflammatory pathways and increased oxidative stress. Moreover, the virus enters the cells by utilizing ACE2 receptors, which are widely expressed in the kidneys. Antioxidant therapy may have potential as a defense against COVID-19 complications in patients with diabetes. Understanding the interaction between SARS-CoV-2 and kidney cells, including the role of ACE2 and miRNAs, is important for future research and therapeutic development. Overall, these findings highlight the importance of comprehensive care and monitoring of kidney health in COVID-19 patients, especially those with underlying kidney conditions.

### Limitations

The authors identified some limitations in this systematic review. First, the inclusion of only English studies may have introduced language bias and potentially excluded relevant studies published in other languages. Second, the reliance on existing systematic reviews as a source for identifying relevant primary articles may have introduced selection bias. Additionally, the authors acknowledged that the search strategy might have missed some relevant studies despite their efforts to conduct a comprehensive literature search.

## Ethical approval

Since this is a systematic review, ethical approval was not required.

## Consent

N/A

## Sources of funding

N/A

## Author contribution

S.A., M.Z., R.A., S.A., and L.A. searched multiple databases, included eligible studies, and extracted data. All authors participated in writing the manuscript and approved the final version.

## Conflicts of interest disclosure

N/A

## Research registration unique identifying number (UIN)

This study is registered in the International Prospective Register of Systematic Reviews (PROSPERO) statement (ID: CRD42015020275).

## Guarantor

All authors: Samar M. Altoukhi, Mariam M. Zamakah, Reman Alharbi, Shatha K. Alghamdi, Lama S. Aldawsari, Hisham Rizk, Muyassar Tarabulsi, Yousif Sandokji.

## Data availability statement

N/A.

## Provenance and peer review

Not commissioned, externally peer-reviewed.

## References

[1] Coronavirus disease (COVID-19) – World Health Organization [Internet] . Accessed February 17, 2024. https://www.who.int/emergencies/diseases/novel-coronavirus-2019

[2] Karimi H Sarmadian R Gilani A . Cerebrovascular accident in a child with precursor B-cell acute lymphoblastic leukemia and coronavirus disease 2019: a case report. J Med Case Rep 2022;16:452. 36471442 10.1186/s13256-022-03672-5 PMC9724282

[3] Gilani A Hajebi Khaniki S Fard F . Comparison of the effects of different COVID-19 vaccine platforms on the hospitalization rate. Vaccine Res 2023;9:42–46.

[4] Alicic RZ Rooney MT Tuttle KR . Diabetic kidney disease: challenges, progress, and possibilities. Clin J Am Soc Nephrol CJASN 2017;12:2032–2045. 28522654 10.2215/CJN.11491116 PMC5718284

[5] Abdulaziz Al-Muhanna F Ibraham Ali Albakr W Subbarayalu AV . Impact of COVID-19 on kidney of diabetic patients. Med Kaunas Lith 2022;58:644. 10.3390/medicina58050644 PMC9143731 35630061

[6] Basra R Whyte M Karalliedde J . What is the impact of microvascular complications of diabetes on severe COVID-19? Microvasc Res 2022r;140:104310. 34979154 10.1016/j.mvr.2021.104310 PMC8719364

[7] D'Marco L Puchades MJ Romero-Parra M . Diabetic kidney disease and COVID-19: the crash of two pandemics. Front Med 2020;7:199. 10.3389/fmed.2020.00199 PMC7218924 32435649

[8] Gilbert RE Caldwell L Misra PS . Overexpression of the Severe Acute Respiratory Syndrome Coronavirus-2 Receptor, Angiotensin-Converting Enzyme 2, in Diabetic Kidney Disease: Implications for Kidney Injury in Novel Coronavirus Disease 2019. Can J Diabetes 2021;45:162–166.e1. 32917504 10.1016/j.jcjd.2020.07.003 PMC7368650

[9] Menon R Otto EA Sealfon R . SARS-CoV-2 receptor networks in diabetic and COVID-19-associated kidney disease. Kidney Int 2020;98:1502–1518. 33038424 10.1016/j.kint.2020.09.015 PMC7543950

[10] Yang Y Zou S Xu G . An update on the interaction between COVID-19, vaccines, and diabetic kidney disease. Front Immunol 2022;13:999534. 36341356 10.3389/fimmu.2022.999534 PMC9630353

[11] Giannini G Carlos Q Velez J May RM . Renal prognosis of COVID-19 associated nephropathy. Kidney Int Rep 2022;7:2722–2725. 36277848 10.1016/j.ekir.2022.09.027 PMC9579006

[12] Paul AK Hossain MK Mahboob T . Does oxidative stress management help alleviation of covid-19 symptoms in patients experiencing diabetes? nutrients 2022;14:321. 35057501 10.3390/nu14020321 PMC8780958

[13] Mourad D Azar NS Azar ST . Diabetic nephropathy and COVID-19: the potential role of immune actors. Int J Mol Sci 2021;22:7762. 34360529 10.3390/ijms22157762 PMC8346171

[14] Sonkar G Singh S Sonkar S . A systematic review approach in understanding the COVID-19 mechanism in diabetes and its progression to diabetic microvascular complications. J Diabetol 2022;13:322.

[15] Srivastava SP Srivastava R Chand S . Coronavirus disease (COVID)-19 and diabetic kidney disease. Pharmaceuticals 2021;14:751. 34451848 10.3390/ph14080751 PMC8398861

[16] Leon-Abarca JA Memon RS Rehan B . The impact of COVID-19 in diabetic kidney disease and chronic kidney disease: a population-based study. Acta Bio-Medica Atenei Parm 2020;91:e2020161. 10.23750/abm.v91i4.10380 PMC7927495 33525210

[17] Bronson SC . Practical scenarios and day-to-day challenges in the management of diabetes in COVID-19 - Dealing with the “double trouble.” Prim Care Diabetes 2021;15:737–739. 34039524 10.1016/j.pcd.2021.05.007 PMC8139261

[18] Schiller M Solger K Leipold S . Diabetes-associated nephropathy and obesity influence COVID-19 outcome in type 2 diabetes patients. J Community Hosp Intern Med Perspect 2021;11:590–596. 34567446 10.1080/20009666.2021.1957555 PMC8462845

[19] Naicker S Yang CW Hwang SJ . The novel Coronavirus 2019 epidemic and kidneys. Kidney Int 2020;97:824–828. 32204907 10.1016/j.kint.2020.03.001 PMC7133222

[20] Li Z Wu M Guo J . Caution on Kidney Dysfunctions of 2019-nCoV Patients [Internet]. medRxiv 2020;2:20021212.

[21] Yamagishi SI Matsui T Nakamura K . Kinetics, role and therapeutic implications of endogenous soluble form of receptor for advanced glycation end products (sRAGE) in diabetes. Curr Drug Targets 2007;8:1138–1143. 17979674 10.2174/138945007782151298

[22] Nomura H Kuruppu S Rajapakse NW . Stimulation of angiotensin converting enzyme 2: a novel treatment strategy for diabetic nephropathy. Front Physiol 2021;12:813012. 35087423 10.3389/fphys.2021.813012 PMC8787214

[23] Malek V Suryavanshi SV Sharma N . Potential of renin-angiotensin-aldosterone system modulations in diabetic kidney disease: old players to new hope!. Rev Physiol Biochem Pharmacol 2021;179:31–71. 32979084 10.1007/112_2020_50

[24] Chen R Lan Z Ye J . Cytokine storm: the primary determinant for the pathophysiological evolution of COVID-19 deterioration. Front Immunol 2021;12:589095. 33995341 10.3389/fimmu.2021.589095 PMC8115911

[25] Zheng T Liu Y Qin S . Increased plasma dipeptidyl peptidase-4 activities are associated with high prevalence of diabetic nephropathy in Chinese patients with newly diagnosed type 2 diabetes: a cross-sectional study. Diab Vasc Dis Res 2016;13:127–136. 26821795 10.1177/1479164115615356

[26] Humpert PM Djuric Z Kopf S . Soluble RAGE but not endogenous secretory RAGE is associated with albuminuria in patients with type 2 diabetes. Cardiovasc Diabetol 2007;6:9. 17343760 10.1186/1475-2840-6-9 PMC1821011

[27] Nakashima S Matsui T Takeuchi M . Linagliptin blocks renal damage in type 1 diabetic rats by suppressing advanced glycation end products-receptor axis. Horm Metab Res Horm Stoffwechselforschung Horm Metab 2014;46:717–721. 10.1055/s-0034-1371892 24710699

[28] Sakata K Hayakawa M Yano Y . Efficacy of alogliptin, a dipeptidyl peptidase-4 inhibitor, on glucose parameters, the activity of the advanced glycation end product (AGE) - receptor for AGE (RAGE) axis and albuminuria in Japanese type 2 diabetes. Diabetes Metab Res Rev 2013;29:624–630. 23861159 10.1002/dmrr.2437

[29] Sultan RH Abdallah M Ali TM . The associations between cytokine levels, kidney and heart function biomarkers, and expression levels of angiotensin-converting enzyme-2 and neuropilin-1 in COVID-19 patients. Vaccines 2022;10:1045. 35891209 10.3390/vaccines10071045 PMC9316107

[30] Sultan RH Elesawy BH Ali TM . Correlations between kidney and heart function bioindicators and the expressions of toll-like, ACE2, and NRP-1 receptors in COVID-19. Vaccines 2022;10:1106. 35891270 10.3390/vaccines10071106 PMC9319872

[31] Loeffler I Rüster C Franke S . Erythropoietin ameliorates podocyte injury in advanced diabetic nephropathy in the db/db mouse. Am J Physiol Renal Physiol 2013;305:F911–F918. 23825071 10.1152/ajprenal.00643.2012

[32] Bondeva T Wojciech S Wolf G . Advanced glycation end products inhibit adhesion ability of differentiated podocytes in a neuropilin-1-dependent manner. Am J Physiol Renal Physiol 2011;301:F852–F870. 21734098 10.1152/ajprenal.00575.2010

[33] Alnomasy SF . Virus-receptor interactions of SARS-CoV-2 spikereceptor-binding domain and human neuropilin-1 b1 domain. Saudi J Biol Sci 2021;28:3926–3928. 33850424 10.1016/j.sjbs.2021.03.074 PMC8032480

[34] Stafford EG Riviere JE Xu X . Pharmacovigilance in patients with diabetes: a data-driven analysis identifying specific RAS antagonists with adverse pulmonary safety profiles that have implications for COVID-19 morbidity and mortality. J Am Pharm Assoc JAPhA 2020;60:e145–e152. 10.1016/j.japh.2020.05.018 PMC7262497 32561317

[35] Sarangarajan R Winn R Kiebish MA . Ethnic prevalence of angiotensin-converting enzyme deletion (D) polymorphism and COVID-19 risk: rationale for use of angiotensin-converting enzyme inhibitors/angiotensin receptor blockers. J Racial Ethn Health Disparities 2021;8:973–980. 32901433 10.1007/s40615-020-00853-0 PMC7478439

[36] Heyman SN Walther T Abassi Z . Angiotensin-(1-7)—a potential remedy for AKI: insights derived from the COVID-19 pandemic. J Clin Med 2021;10:1200. 33805760 10.3390/jcm10061200 PMC8001321

[37] Kant S Menez SP Hanouneh M . The COVID-19 nephrology compendium: AKI, CKD, ESKD and transplantation. BMC Nephrol 2020;21:449. 33109103 10.1186/s12882-020-02112-0 PMC7590240

[38] Chu KH Tsang WK Tang CS . Acute renal impairment in coronavirus-associated severe acute respiratory syndrome. Kidney Int 2005;67:698–705. 15673319 10.1111/j.1523-1755.2005.67130.x PMC7112337

[39] Werion A Belkhir L Perrot M . SARS-CoV-2 causes a specific dysfunction of the kidney proximal tubule. Kidney Int 2020;98:1296–1307. 32791255 10.1016/j.kint.2020.07.019 PMC7416689

[40] Rivero J Merino-López M Olmedo R . Association between postmortem kidney biopsy findings and acute kidney injury from patients with SARS-CoV-2 (COVID-19). Clin J Am Soc Nephrol CJASN 2021;16:685–693. 33782033 10.2215/CJN.16281020 PMC8259494

[41] Fani M Zandi M Ebrahimi S . The role of miRNAs in COVID-19 disease. Future Virol 2021;16:301–306.

[42] D’Marco L Puchades MJ Serra MÁ . SARS-CoV-2 vs. hepatitis virus infection risk in the hemodialysis population: what should we expect? Int J Environ Res Public Health 2021;18:5748. 34071948 10.3390/ijerph18115748 PMC8198690

[43] Widiasta A Sribudiani Y Nugrahapraja H . Potential role of ACE2-related microRNAs in COVID-19-associated nephropathy. Non-Coding RNA Res 2020;5:153–166. 10.1016/j.ncrna.2020.09.001 PMC7480227 32923747

[44] Srivastava SP Goodwin JE Tripathi P . Interactions among long non-coding RNAs and microRNAs influence disease phenotype in diabetes and diabetic kidney disease. Int J Mol Sci 2021;22:6027. 34199672 10.3390/ijms22116027 PMC8199750

[45] Vishnubalaji R Shaath H Alajez NM . Protein coding and long noncoding RNA (lncRNA) transcriptional landscape in SARS-CoV-2 infected bronchial epithelial cells highlight a role for interferon and inflammatory response. Genes 2020;11:760. 32646047 10.3390/genes11070760 PMC7397219

[46] Wei L Li J Han Z . Silencing of lncRNA MALAT1 prevents inflammatory injury after lung transplant ischemia-reperfusion by downregulation of IL-8 via p300. Mol Ther Nucleic Acids 2019;18:285–297. 31604167 10.1016/j.omtn.2019.05.009 PMC6796730

[47] Zhang Q Chen CY Yedavalli VSRK . NEAT1 long noncoding RNA and paraspeckle bodies modulate HIV-1 posttranscriptional expression. mBio 2013;4:e00596–00512. 23362321 10.1128/mBio.00596-12 PMC3560530

[48] Wen S Li S Li L . circACTR2: a novel mechanism regulating high glucose-induced fibrosis in renal tubular cells via pyroptosis. Biol Pharm Bull 2020;43:558–564. 32115515 10.1248/bpb.b19-00901

[49] Rao S Ali K Dennis J . Analysis of glucose levels in patients hospitalized with COVID-19 during the first phase of this pandemic in West Texas. J Prim Care Community Health 2020;11:2150132720958533. 32924762 10.1177/2150132720958533 PMC7493238

[50] Sidorenkov G Navis G . Safety of ACE inhibitor therapies in patients with chronic kidney disease. Expert Opin Drug Saf 2014;13:1383–1395. 25148900 10.1517/14740338.2014.951328

[51] Wan Y Shang J Graham R . Receptor recognition by the novel Coronavirus from Wuhan: an analysis based on decade-long structural studies of SARS Coronavirus. J Virol 2020;94:e00127–20. 31996437 10.1128/JVI.00127-20 PMC7081895

[52] Mohamed NE Benn EKT Astha V . Association between chronic kidney disease and COVID-19-related mortality in New York. World J Urol 2021;39:2987–2993. 33481113 10.1007/s00345-020-03567-4 PMC7821175

